# The effects of currents and potentials on the selectivities of copper toward carbon dioxide electroreduction

**DOI:** 10.1038/s41467-018-03286-w

**Published:** 2018-03-02

**Authors:** Dan Ren, Jinhuan Fong, Boon Siang Yeo

**Affiliations:** 1 Department of Chemistry, Faculty of Science, National University of Singapore, 3 Science Drive 3, Singapore, 117543 Singapore; 2 Solar Energy Research Institute of Singapore (SERIS), National University of Singapore, 7 Engineering Drive 1, Singapore, 117574 Singapore

## Abstract

Copper electrodes have been shown to be selective toward the electroreduction of carbon dioxide to ethylene, carbon monoxide, or formate. However, the underlying causes of their activities, which have been attributed to a rise in local pH near the surface of the electrode, presence of atomic-scale defects, and/or residual oxygen atoms in the catalysts, etc., have not been generally agreed on. Here, we perform a study of carbon dioxide reduction on four copper catalysts from −0.45 to −1.30 V vs. reversible hydrogen electrode. The selectivities exhibited by 20 previously reported copper catalysts are also analyzed. We demonstrate that the selectivity of carbon dioxide reduction is greatly affected by the applied potentials and currents, regardless of the starting condition of copper catalysts. This study shows that optimization of the current densities at the appropriate potential windows is critical for designing highly selective copper catalysts.

## Abstract (web summary)

Electrochemical reduction of carbon dioxide provides a means to convert environmental carbon into utile products. Product selectivity factors are disputed. Here, authors examine 24 copper catalysts and show that mass transport plus the electrochemical potential window drive selectivity.

## Introduction

The reduction of carbon dioxide (CO_2_) to fuels and chemical feedstocks using renewable electricity has the potential of becoming a key component in the development of a sustainable carbon cycle^1,2^. This process requires selective, efficient, and stable electrocatalysts before it can be implemented in the industrial scale. Among the metal electrocatalysts studied, copper is the only metal that can reduce CO_2_ to significant amounts of hydrocarbons and oxygenates^3,4^. Of these products, methods to selectively form formate (HCOO^−^)^5^, carbon monoxide (CO)^6^, methane (CH_4_)^7^, ethylene (C_2_H_4_)^8–10^ and ethanol (C_2_H_5_OH)^11^ have been extensively pursued.

To understand how these products are formed on copper, Peterson and Nørskov modeled the reduction of CO_2_ on Cu(211) surface (a stepped surface) using density functional theory (DFT)^12^. As the applied potential became more negative, CO_2_ was first reduced to HCOO^−^ and CO, from −0.41 V vs. RHE (reversible hydrogen electrode) onward. C_2_H_4_ and CH_4_ were the next major products and evolved at potentials negative to −0.71 V vs. RHE. These theoretical predictions are consistent with the findings reported earlier by Hori et al. on a polycrystalline Cu surface, where HCOO^−^ and CO were produced first, followed by C_2_H_4_ and CH_4_^13^. Interestingly, when Cu single-crystal surfaces were studied using chronopotentiometry at −5 mA cm^−2^ in 0.1 M KHCO_3_ electrolyte, the selectivity between C_2_H_4_ and CH_4_ changed. Cu(100) and Cu(111) were, respectively, found selective for C_2_H_4_ and CH_4_ formation. Further optimization of C_2_H_4_ production could be achieved by using stepped-Cu(100) surfaces^14^. For example, the faradaic efficiency (FE) of C_2_H_4_ reached 50%, while CH_4_ was suppressed to 5% on a Cu(S)-[4(100)×(111)] electrode polarized at −5 mA cm^−2^ (−0.94 V vs. RHE). Oddly and still without an explanation, the selectivity of neither C_2_H_4_ nor CH_4_ seemed to improve significantly on stepped-Cu(111) surfaces.

Besides the Cu(100) single-crystal series, some oxide-derived Cu catalysts have also shown a propensity for C_2_H_4_ and C_2_H_5_OH production^8–10,15,16^. For example, we have found that at −0.99 V vs. RHE, Cu films reduced from μm-thick Cu_2_O could catalyze the reduction of CO_2_ in 0.1 M KHCO_3_ electrolyte to C_2_H_4_ with FEs = 34–39%, while the FEs of CH_4_ were minimized to <1%^9^. Interestingly, thick oxide-derived Cu films, which do not appear to have very different morphological and chemical differences as the aforementioned C_2_-selective catalysts, have also been independently reported to be selective toward the formation of CO and HCOO^−^^5,6^. The selective reduction of CO_2_ to CH_4_ on Cu surfaces has been relatively less studied. Recently, Manthiram et al. reported that 7 nm-sized Cu nanoparticles dispersed on a glassy carbon electrode could catalyze the formation of CH_4_ with an average FE of 80% in 0.1 M NaHCO_3_ at −1.25 V vs. RHE^7^.

Based on characterizations of the catalysts presented in the above reports, the underlying causes for the selective reduction of CO_2_ to various products on Cu catalysts have been attributed to factors such as morphology^9^, particle sizes^17,18^, crystallite sizes and facets (for example, Cu(100))^18^, grain boundaries^19^, strains^20^, presence of residual oxygen (or Cu^+^)^10,21^, and rise in local pH at the surface of the electrode^8^. However, the effect of applied potential on the selectivity of these catalysts, as shown earlier by Peterson and Nørskov has been largely neglected or sidestepped in these important works^12^.

Here, we address this inadequacy by studying a series of Cu catalysts (metallic film and oxide-derived films) with different roughness factors for the electroreduction of CO_2_. We find that the selectivities of CO_2_ reduction toward HCOO^−^/CO, C_2_H_4_, and CH_4_ are observed to occur at different potential windows, as long as the mass transport limitation of CO_2_ is not reached. Furthermore, this finding can be used to explain the selectivities exhibited by a range of Cu single-crystal surfaces and nanostructures. We also discuss the role of oxide in oxide-derived Cu catalysts for the enhanced formation of CO, HCOO^−^, and C_2_H_4_.

## Results

### Characterization of the catalysts

**Fig. 1:**
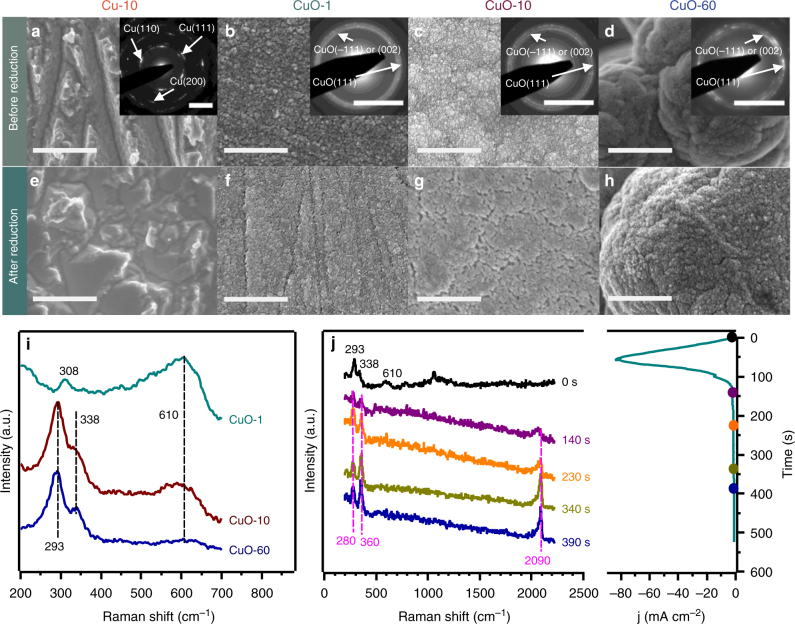
Material characterization of four catalysts. SEM images and SAED patterns (inserts) of (**a**) Cu-10, (**b**) CuO-1, (**c**) CuO-10, and (**d**) CuO-60 catalysts before reduction; SEM images of (**e**) Cu-10, (**f**) CuO-1, (**g**) CuO-10, and (**h**) CuO-60 catalysts after reduction; (**i**) Raman spectra of Cu oxide surfaces collected ex situ and (**j**) operando Raman spectroscopy with the simultaneously obtained chronoamperogram of CuO-60 during CO_2_ reduction in 0.1 M KHCO_3_ at −0.5 V vs. RHE. Scale bars: 1 μm for SEM (**a**–**g**) and 5 nm^−1^ for SAED (inserts of **a**–**d**)

The copper catalysts were prepared via electrodeposition^22^. By tuning the pH of the electrolyte and the deposition time, four different films were deposited (Supplementary Figure 1). These catalysts were characterized by scanning electron microscopy (SEM), selected area electron diffraction (SAED, with transmission electron microscopy, TEM), and Raman spectroscopy (Fig. 1). As shown by their SAED patterns, 10 min depositions using pH = 10.5 electrolyte led to the formation of metallic Cu films (Cu-10), while the other three catalysts deposited using pH = 13.2 electrolyte for 1 min, 10 min, and 60 min were all CuO (termed as CuO-1, CuO-10, and CuO-60, respectively). The morphologies of the four films before and after CO_2_ reduction were revealed by SEM. The surface of Cu-10 consisted of μm-sized particles before reduction (Fig. 1a). The surfaces of CuO-1, CuO-10, and CuO-60 were covered with 10–100 nm particles, though the nanoparticles of CuO-60 agglomerated as μm-sized particles (Fig. 1b–d). After reduction, there was no significant morphology change for all four catalysts (Fig. 1e–h). The roughness factors (RFs) of post-reduced Cu-10, CuO-1, CuO-1, and CuO-60 were estimated by the double layer capacitance method to be 1.4, 5, 48, and 186, respectively (Supplementary Figure 2 and Supplementary Table 1).

The Raman spectra of the three CuO films exhibited peaks at 293, 308, 338, and 610 cm^−1^, which could be assigned to signals from CuO (Fig. 1i)^22,23^. According to the Pourbaix diagram of the Cu-H_2_O system, the three CuO samples would be reduced to the metallic state under CO_2_ reduction potentials, i.e., potentials more negative than −0.50 V vs. RHE (all potentials cited in this work are referenced to the RHE)^24^. This was further verified by operando Raman spectroscopy of the catalysts (representative data for CuO-60 in Fig. 1j). Upon application of an electrochemical potential of −0.50 V vs. RHE, the 293, 338, and 610 cm^−1^ peaks of CuO disappeared within 140 s. From 140 s onward, three bands at 280, 360, and 2090 cm^−1^ appeared. These can be, respectively, assigned to the restricted rotation of bound CO, the Cu-CO stretching and C≡O stretching modes^25–27^. These bands were also observed on CuO-60 at more negative potentials from −0.60 to −0.80 V (Supplementary Figure 3), where hydrocarbons such as C_2_H_4_ were formed with appreciable quantities (FE_ethylene_ = 3–19%). This observation is consistent with previous reports that indicates CO is a key intermediate formed during the reduction of CO_2_ to hydrocarbons on Cu catalysts^13,28^. The appearance of Cu–CO signals only after the CuO signals have disappeared indicates that the active sites for CO_2_ reduction are likely to be metallic Cu. The reduction of oxide is also indicated by the chronoamperogram, which showed a reduction peak in the first 100 s (Fig. 1j).

### The effects of limiting currents and applied potentials on CO_2_ reduction activity

The electrochemistry of our four catalysts toward CO_2_ reduction was assessed using 60 min chronoamperometry in aqueous 0.1 M KHCO_3_ electrolyte. A three-electrode setup^29^ was used and the products were quantified using gas chromatography and high performance liquid chromatography^2^.

**Fig. 2:**
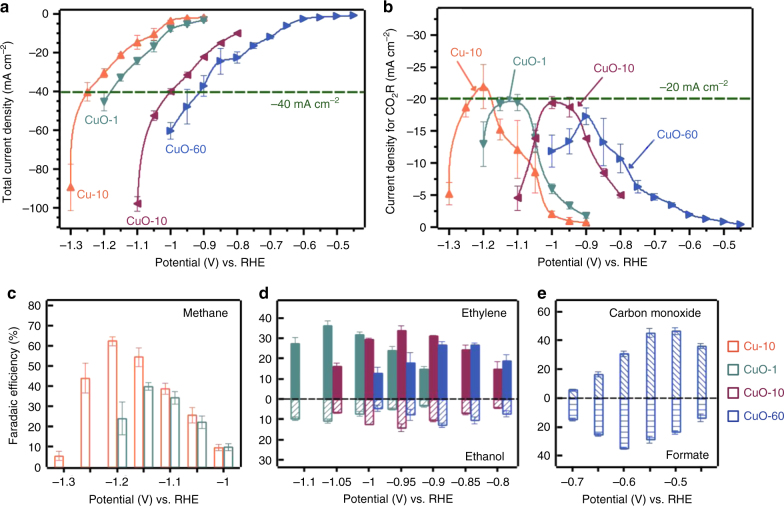
Electrocatalytic performance of four catalysts toward carbon dioxide electroreduction. **a** Total geometric current density and **b** current density for CO_2_ reduction (CO_2_R) on four catalysts at different potentials; **c** faradaic efficiency of methane on Cu-10 and CuO-1 catalysts at different potentials; **d** faradaic efficiency of ethylene and ethanol on CuO-1, CuO-10, and CuO-60 catalysts at different potentials; **e** faradaic efficiency of carbon monoxide and formate on CuO-60 catalyst at different potentials. Error bars in **a**–**e** represent the standard deviations of three independent measurements

The total current density and CO_2_ reduction current density are presented in Fig. 2a, b. In general, the total current densities exhibited by the catalysts correlated positively with the latters’ roughness factors and with the overpotentials applied. However, the CO_2_ reduction current densities on the four catalysts exhibited parabolic-like trends against the applied potentials, with maxima of about −20 mA cm^−2^. These maxima corresponded to total current densities of about −40 mA cm^−2^. Beyond the limiting total current density, the CO_2_ reduction current densities decreased and the selectivity toward H_2_ production increased. The limiting CO_2_ current can be attributed to the mass transport limitation of CO_2_ to the electrode, as CO_2_ has a poor solubility in aqueous electrolytes (~34 mM at 25 °C). Additionally, as the total current increased with applied overpotentials, there will also be a buildup of OH^−^ near the electrode surface, i.e., an increase in local pH. This will cause a decrease in local concentration of CO_2_ near the electrode surface, resulting in the fall in CO_2_ reduction currents^30,31^. These findings are supported by numerical simulations of the concentrations of CO_2_ and local pH values at the Cu surface (using Cu-10 as the model; Supplementary Figure 4). As the simulated current increased to −90 mA cm^−2^, the local pH increased from a bulk value of 6.8 to 11.5, while the local CO_2_ concentration fell from 34 mM to 6.5 mM ^32^.

The FEs of major CO_2_ reduction products are presented in Fig. 2c–e (Supplementary Tables 2-5). We found that a slightly roughened metallic Cu surface (Cu-10, RF = 1.4) exhibited excellent CH_4_ selectivity at −1.2 V with FE_methane_ of 62%. The oxide-derived Cu surfaces (CuO-1, RF = 5; CuO-10, RF = 48, and CuO-60, RF = 186) were generally more selective for C_2_H_4_ and C_2_H_5_OH (up to total FE of 48%)^9,19^. Interestingly, CuO-1 also exhibited a good selectivity toward CH_4_ at −1.15 V with a FE of 40%. This observation appears unusual as oxide-derived Cu films are hitherto not known to be selective toward the formation of CH_4_^5,9^. We also note that the roughest sample, CuO-60, gave relatively high selectivities for CO and HCOO^−^ at low overpotentials (FE_CO_ = 46% at −0.50 V and FE_formate_ = 35% at −0.60 V).

Another observation is that the catalytic selectivity toward the formation of different products was only observed in specific potential ranges. At potentials more positive than −0.7 V, CO and HCOO^−^ (maximum total FE = 75% at −0.55 V) were significantly formed. From −0.8 to −1.1 V, C_2_H_4_ and C_2_H_5_OH were produced in great quantities by all the CuO catalysts. As the overpotential increased further (more negative than −1.1 V), CH_4_ was selectively produced (high CH_4_ selectivity on Cu-10 at −1.15 V and CuO-1 at −1.2 V). Only small amounts of CH_4_ were produced using CuO-10 and CuO-60. This is likely because their total current density exceeded the limiting current density of −40 mA cm^−2^ after the potential reached more negative to −1.1 V, which caused significant decrease of the CO_2_ concentration near the electrode.

### Proposed mechanism for the formation of major C_1_ and C_2_ products

**Fig. 3:**
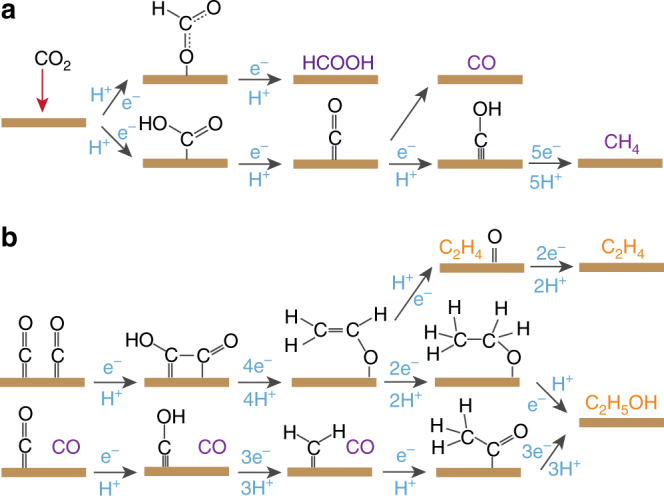
Proposed mechanism for carbon dioxide electroreduction. **a** The pathway to C_1_ products (formate, carbon monoxide, and methane) and **b** the pathway to C_2_ products (ethylene and ethanol). Water molecules are not drawn and the formation of C_2_ products is drawn from carbon monoxide. Purple color indicates a free C_1_ molecule and orange color indicates a free C_2_ molecule

Considering the electrocatalytic properties of different Cu catalysts and the DFT calculations reported in the literature^12,33–36^, we propose a mechanism for the formation of major C_1_ and C_2_ products (Fig. 3). After one proton and one electron transfer, CO_2_ could be reduced to either *OCHO or *COOH intermediate. Further reduction of *OCHO and *COOH lead to the respective formation of HCOO^−^ and *CO. The presence of *CO as an intermediate species of CO_2_ reduction is demonstrated by our operando Raman spectroscopy results in Fig. 1j and Supplementary Figure 3. In agreement with previous studies on a range of Cu catalysts, we found that the potential-dependent profile of HCOO^−^’s faradaic efficiency does not exactly follow that of CO on CuO-60 (Fig. 2e)^4–6,9^. This indicates that CO and HCOO^−^ may not have been formed through the same *COOH intermediate^34,36^. This finding is consistent with recent DFT calculations, which shows that the activities of a range of metallic catalysts toward HCOO^− ^formation correlate better with their binding energies to *OCHO intermediates^36^. *CO could be further reduced to CH_4_, or undergo C–C coupling with another *CO to form C_2_H_4_ and C_2_H_5_OH. It is notable that to-date copper is the only metal electrode capable of facilitating these value-added reactions at reasonable rates as *CO is optimally bonded to it (Cu resides near the top of the volcano plot)^37^. We have also recently proposed a CO-insertion mechanism for the formation of C_2_H_5_OH in a CO rich environment^11^. The different energy barriers in the multiple pathways to form CO, HCOO^−^, C_2_H_4_, C_2_H_5_OH, and CH_4_ could be the reason why these products were observed at different potential windows (Fig. 2c–e)^38^.

## Discussion

**Fig. 4:**
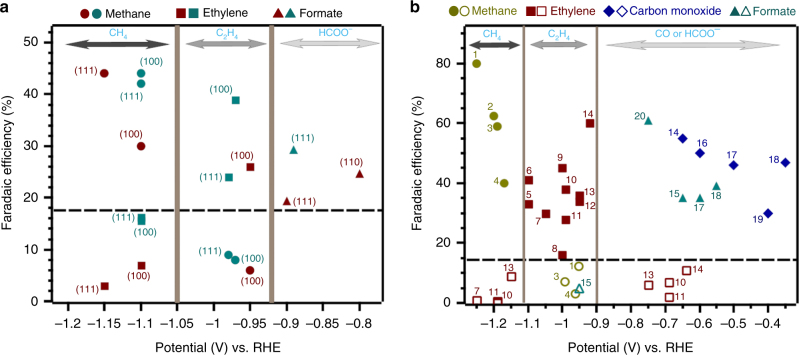
Potential windows for the selective formation of different products. **a** Faradaic efficiency of ethylene, methane, and formate on different copper single crystals at different potentials from ref. ^43^ (wine) and ref. ^44^ (cyan). **b** The selectivities of 20 different Cu catalysts reported by 11 different research groups at different applied potentials. (1) isolated particles—ref. ^7^; (2) Cu-10 in this work; (3) polycrystalline—ref. ^9^ ; (4) polycrystalline—ref. ^4^; (5) Cu_2_O (3 C cm^−2^)—ref. ^8^; (6) 44 nm cubes—ref. ^47^; (7) nanoparticles—ref. ^45^; (8) KF roughened Cu—ref. ^46^; (9) electrochemically cycled Cu—ref. ^31^; (10) Cu_2_O (1.7 μm)—ref. ^9^; (11) mesocrystals—ref. ^40^; (12) CuO-10 in this work; (13) nanocrystals (Cu-NC10)—ref. ^45^; (14) Cu_2_O (O_2_ plasma 20 min—ref. ^10^; (15) nanocrystals (Cu-NC20)—ref. ^45^; (16) CuO nanowire—ref. ^6^; (17) CuO-60 in this work; (18) Cu_2_O (annealing)—ref. ^5^; (19) Cu_2_O (11 C cm^-2^)—ref. ^8^; (20) CuO nanoparticles—ref. ^48^

On the basis of the preceding results, we propose here that the effects of tuning the morphology of a Cu catalyst may not solely lie in creating catalytically active sites (as commonly believed)^9,39–41^, but also affects its roughness so that the material operates with a suitable current density at a particular potential. Here, we discuss this proposition in conjunction with previous works (Fig. 4, Supplementary Tables 6 and 7). In Hori’s classic work, a constant current of −5 mA cm^−2^ was applied during CO_2_ reduction on all the Cu single crystals^14,42^. Under these conditions, Cu(100) and Cu(111) were, respectively, more selective for C_2_H_4_ and CH_4_ formation. Introducing (110) atomic steps to Cu(100) terraces to create high index surfaces was shown to further enhance C_2_H_4_ selectivity. These studies suggest that surface crystallography is paramount in the control of product selectivity. However, two recent CO_2_ reduction studies performed at various potentials (−0.30–−1.25 V by our group and −0.89–−1.15 V by Hahn et al.) revealed that Cu(100) could also reduce CO_2_ to CH_4_ with FE of 30–44% at about −1.1 V (Fig. 4a)^43,44^. It is remarkable that the enhanced selectivities for CH_4_, C_2_H_4_, and HCOO^−^ formation (the formation of CO is generally <15% in these studies) on all the surfaces studied are most pronounced in distinct potential ranges. At potentials negative or equal to −1.1 V, CH_4_ selectivity was enhanced and the faradaic efficiency of C_2_H_4_ was low on Cu(111) and Cu(100). At potentials more positive than −1.0 V, C_2_H_4_ selectivity was observed and the FE of CH_4_ was suppressed to <10%, including on Cu(111). High faradaic efficiencies (FE) of HCOO^−^ were observed at potentials more positive to −0.9 V on single crystals, especially on Cu(110). These evidences indicate that the applied potential is a crucial factor for the selectivities of different Cu single crystals. The corollary is that the frequently generalized statement ‘enhanced CH_4_ selectivity using Cu(111)’ or ‘enhanced C_2_H_4_ selectivity using Cu(100)’ is only true under specific conditions^14,41,42^.

Besides Cu single crystals, we found that the selectivities of 20 other copper catalysts including metallic nanoparticles, oxide-derived nanoparticles, nanorods, etc. were also greatly affected by the applied potentials (Fig. 4b). To achieve a high selectivity of CH_4_ on catalysts, such as isolated Cu nanoparticles^7^, polycrystalline Cu^4,13^, as well as Cu-10 in this study, the applied potential was always more negative than −1.1 V (solid circles). Note that these catalysts have relatively smooth surfaces and as such, do not exhibit current density that exceeds −40 mA cm^−2^ (the limiting current density) at negative potentials ~−1.2 V. For catalysts that favor C_2_H_4_ formation such as Cu_2_O films^8–10^, Cu nanoparticles^45^, Cu nanocubes^31,40,46,47^, Cu nanocrystals^45^, and CuO films in this study, the optimum potential range was from −0.9 to −1.1 V (solid squares). CO and HCOO^−^ selectivity were usually observed on rough and thick oxide-derived Cu films at potentials positive to −0.7 V^5,6,8,10,45,48^.

Hence, the top three regions in Fig. 4b (from left to right) indicates the most suitable potential ranges for the selective formation of CH_4_, C_2_H_4_, and CO/HCOO^−^, which are, respectively, about <−1.1 V, −0.9 V to −1.1 V, and > −0.9 V. It is notable that outside their suitable potential windows, these products are usually produced with FE <15% (hollow shapes in the bottom three regions of Fig. 4b). For example, the FE of C_2_H_4_ using ‘ethylene-selective’ catalysts were <11% at potentials negative to −1.1 V or positive to −0.8 V (hollow squares). We note here that the selectivities of some catalysts could not be clearly defined at the potentials interfacing two potential windows, such as at −0.90 V. For instance, apart from catalyzing the reduction of CO_2_ to C_2_H_4_ at −0.90 V (FE = 15%), CuO-1 could also catalyze the formation of HCOO^−^ with an appreciable faradaic efficiency of ~20%. However, its selectivity toward HCOO^−^ is lower compared with that of the thicker films (HCOO^−^ forms at a FE of 35 % on CuO-60 at −0.60 V).

The potential windows highlighted in Fig. 4 are applicable to reports where the surfaces of the catalysts were particulate or planar, the electrolysis cell was similar to the design of Kuhl et al^4^ and the electrolyte was aqueous 0.1 M KHCO_3_ or other similar electrolytes (such as 0.1 M NaHCO_3_). Studies using significantly different cell designs^49^, gas diffusion electrodes^16^, nanoneedles, or nanofoams^50^ may exhibit different potential ranges for a specific product since these systems may have higher limiting current densities. The use of KOH or KCl electrolytes^16,51^ may introduce other effects such as high local pH, and thus, do not fit properly in the above-defined regions.

The aforementioned potential windows (Fig. 4) also show that the DFT predictions made by Peterson and Nørskov on how potentials affect product selectivity is not only applicable to Cu(211) surfaces^12^, but can also be applied to a variety of Cu catalysts such as Cu single crystals, oxide-derived Cu and Cu nanostructures, as long as mass transport limitation of CO_2_ has not been reached.

It is interesting that CuO-1, an oxide, exhibited a surprisingly high faradaic efficiency of CH_4_ (Fig. 2c). In fact, from −0.95 to −1.15 V, the FE of CH_4_ on CuO-1 (3–40%) and Cu-10 (2–54%) are comparable. This observation is remarkable because oxidized Cu catalysts are known for their propensity to reduce CO_2_ to C_2_ products, rather than to CH_4_^8–10^. Hence, this result indicates that the presence of oxide is not the most crucial factor in determining selectivity between CH_4_ and C_2_H_4_. We further note that an electropolished Cu surface would be oxidized once exposed to air (which is almost inevitable during sample transfer) and yet, this catalyst is known to produce high FE of CH_4_ at negative potentials (Fig. 4b)^4^.

An insight could also be gained from the selectivities exhibited by oxide-derived Cu catalysts reported by many groups, including Kanan, Baltrusaitis, and ours^5,6,8,9,31,48^. These films have been reported selective toward the reduction of CO_2_ to either HCOO^−^ or C_2_–C_3_ products. This behavior is intriguing if we consider that the chemical identities and morphologies (in the nanometer scale) of these films do not appear very different^8^. For example, using a thick layer of Cu oxide (prepared by annealing a Cu foil at 500 °C for 12 hours), Kanan et al. reported FEs of 47% for CO and 39% for HCOO^−^ at −0.35 and −0.55 V, respectively. C_2_H_4_ was formed at a maximum FE of only ~5% at −0.95 V^5^. The production of C_2_H_4_ is, thus, notably lower than that on a polycrystalline Cu surface (FE = 23% at −0.97 V) and our oxide-derived Cu films (34–39% at −0.99 V)^9,13^. The fact that Kanan’s Cu sample exhibited rather low selectivities toward C_2_H_4_ seem to contradict with observations made by Baltrusaitis et al. and our group. We propose here a simple explanation for this observation: apart from the slightly different electrolytes used, we highlight that the high total current density (about −25 mA cm^−2^) exhibited by Kanan’s annealed Cu at −0.95 V will result in a lower local concentration of CO_2_ near the electrode surface. This will cause an overall lowering of the FE for CO_2_ reduction and consequently, a decreased formation of C_2_H_4_ at −0.95 V. Our observation indicates that thick oxide films will not be suitable for C_2_H_4_/C_2_H_5_OH formation, once their roughness factors exceed the optimum^9^.

Schouten and Koper have measured the onset potentials required for the formation of CH_4_ and C_2_H_4_ during CO reduction on Cu(100) and Cu(111) electrodes^52,53^. It was shown that C_2_H_4_ could be formed at ~400 mV lower overpotential compared to CH_4_ on Cu(100) in pH = 7 electrolyte. C_2_H_4_ was further proposed to be formed via a CO dimerization pathway on Cu(100). Although this study had revealed the potential-dependence of C_2_H_4_ formation on Cu(100), we draw here a more general trend of how the electrochemical potential affects the selectivity of CO_2_ reduction for a wide range of Cu surface structures. The effect of limiting current density on selectivities is also addressed here.

Finally, our work highlights how we could design Cu catalysts with various selectivities—it is critical to control the surface roughness such that the limiting current density lies in the suitable potential window for CH_4_, C_2_H_4_, or CO/HCOO^−^. In order to achieve a high selectivity of CH_4_ formation, the catalyst should be relatively smooth so that the limiting current density is not exceeded at the very negative applied potentials needed (i.e., more negative than −1.2 V). With a roughness factor of 1.4, Cu-10 catalyst exhibits both high faradaic efficiency (62%) and high partial current density (−18 mA cm^−2^) of CH_4_, making it among the best catalysts toward CH_4_ formation^7^. For C_2_H_4_ and C_2_H_5_OH selectivity, the catalyst should have a slightly roughened surface so that the intermediates are stabilized and its CO_2_ reduction current density is maximized at regions from −0.9 V to −1.1 V. To obtain a high faradaic efficiency of CO or HCOO^−^, the catalysts should be thick and rough oxide-derived films with high RFs. We note here that though smooth surfaces such as Cu(110), polycrystalline Cu, and Cu-10 could catalyze the reduction of CO_2_ to HCOO^−^ or CO at more negative potentials, their selectivities are not comparable with those exhibited by thick Cu oxide films at less negative potentials^13,43^.

In this work, we prepared four Cu-based films by electrodeposition, and showed that they exhibited different selectivities toward CO_2_ reduction at different potential ranges. We highlight how limiting currents and applied potentials affect the selectivity of CO_2_ reduction reactions. This helps to rationalize the selectivity of different Cu catalysts, not only in this work, but also from many other reports. A FE of ~40% of CH_4_ observed on CuO-1 film at −1.15 V contradicts with a proposition that Cu^+^ or subsurface oxygen-modified Cu is the preferred catalyst for C_2_H_4_ selectivity. This study strongly shows that optimization of CO_2_ reduction current densities at the appropriate potential windows is critical for forming the type of product needed, and thus provides us with insights into how highly selective Cu catalysts could be rationally designed.

## Methods

### Preparation of electrocatalysts

The substrates were mechanically polished Cu discs (99.99%, Goodfellow) with an exposed geometric surface area of 0.865 cm^2^. Two aqueous deposition electrolytes, A and B were prepared, for the depositions of metallic and oxide films, respectively.

Electrolyte A was prepared by first dissolving tartaric acid (Alfa Aesar, 99%) in deionized water (18.2 MΩ cm, Barnstead Type 1). CuSO_4_ 5H_2_O (GCE, 99%) was then added. The solution was then cooled and continuously stirred in an ice water bath while NaOH (Chemicob, 99%) was slowly added. The final pH of the solution was 10.5, with the concentrations of tartaric acid, CuSO_4_ 5H_2_O and NaOH at, respectively, 0.2, 0.2, and 0.8 M. The deposition of Cu-10 was carried out in a two-electrode setup with a Pt wire as the counter electrode. Chronopotentiometry at 8 mA cm^−2^ for 10 min was used for the deposition of Cu-10.

Electrolyte B was prepared similarly as above except that it had 2.5 M NaOH, resulting in a pH 13.2 solution. The deposition of CuO-1 was carried out in a three-electrode setup with a Pt wire and a Ag/AgCl (Saturated KCl, Pine) as the counter and reference electrodes, respectively. Chronamperometry at 1.47 V vs. RHE for 1 min (Autolab PGSTAT30) was used for the deposition of CuO-1. The deposition of CuO-10 and CuO-60 were carried out in a two-electrode setup with a Pt wire as the counter electrode. CuO-10 and CuO-60 were deposited by applying 8 mA cm^−2^ for 10 min and 60 min, respectively[22].

### Characterization of the electrocatalysts

The chemical compositions of the catalysts were characterized using SAED (SAED, TEM mode, JEOL 3010, 300 kV, 112 μA) and Raman spectroscopy (Modular System, Horiba Jobin Yvon). A He–Ne laser was used as the excitation source and the acquisition time was 10 seconds for each spectrum. A dry objective (Olympus MPlan N, 50×, numerical aperture = 0.75) and a water immersion objective (Olympus LUMFL, 60×, numerical aperture = 1.10) were, respectively, employed for ex situ and operando Raman spectroscopy. The morphologies of the catalysts were characterized by SEM (JEOL JSM-6710F, 5 kV). Their electrochemical-active surface areas were determined by their double layer capacitances in N_2_-saturated 0.1 M KClO_4_ (99.9%, Sigma Aldrich). A three-electrode setup was used with a Pt wire counter and a Ag/AgCl reference electrode (Saturated KCl, Pine). Cyclic voltammetry were performed in a non-faradaic region from −0.05 V to 0.05 V vs. RHE. The scan rates were 50, 100, 150, 200, 250, 300, 350, and 400 mV s^−1^.

### Electrochemical reduction of CO_2_

CO_2_ electroreduction was performed in a custom built three-electrode electrochemical cell^45^. The reference and counter electrodes were, respectively, a Ag/AgCl (saturated KCl, Pine) and a coiled Pt wire. The cathodic and anodic compartments were separated by an anion exchange membrane (Asahi Glass). Both compartments were filled with CO_2_-saturated 0.1 M KHCO_3_ (99.7%, Merck). During 60 min chronoamperometry, 20 cm^3^ min^−1^ of CO_2_ was continuously flowed into the electrolyte. The gas outlet of cathodic compartment was connected to a gas chromatograph (GC, Agilent 7890A) for the online quantification of gas products. The liquid products were quantified by headspace gas chromatography (HS-GC, Agilent 7890B) and high performance liquid chromatography (HPLC, Agilent 1260) after electrolysis. The voltage drop was automatically compensated using the current-interrupt mode available in the potentiostat (Gamry 600). The voltage was converted to the RHE scale and the current density was normalized to the exposed geometric surface area. All the detected products were quantified in terms of their FE. The FE of product *X* is defined as:

$$\mathrm{FE}\left( X \right) = \frac{{\mathrm{Number}\;{\mathrm {of}\;{\mathrm {electrons}}\;{\mathrm {used}}\;{\mathrm {for}}\;{\mathrm {producing}}\;X}}}{{\mathrm {Total}\;{\mathrm {number}\;{\mathrm {of}}\;{\mathrm {electrons}}\;{\mathrm {used}}\;{\mathrm {for}}\;{\mathrm {electrolysis}}}}} \times 100{\mathrm{\% }}$$

Each reported FE value was the average of three independent sets of measurements.

### Numerical simulations of local pH and CO_2_ concentration

Numerical simulations were performed with MATLAB 8.5 to calculate the local pH and how the concentration of CO_**2**_ near the electrode changed as a function of current density^32^. The electrode was taken to be planar (1-D model). The corresponding bulk concentration of CO_2_, HCO_3_^−^, CO_3_^2−^, and H^+^, diffusion coefficients, and rate constants were taken from the work of Gupta et al., along with the boundary conditions^32^. The diffusion layer thickness was assumed to be 36 μm^54^. The normalized current density (against the electrochemical-active surface area) and the FE were used for calculations^18^. Only the effect of diffusion and surface reaction were considered in this model. Only the Cu-10 surface was simulated as it has a small roughness factor of 1.4, and, thus, its surface is close to planar.

### Data availability

The data that support the findings of this study are available from the corresponding authors.

## Electronic supplementary material

Supplementary Information
