# Supplementary material for: The effects of currents and potentials on the selectivities of copper toward carbon dioxide electroreduction
Source: Nat Commun. 2018 Mar 2;9:925. doi: 10.1038/s41467-018-03286-w (PMC5834446; doi:10.1038/s41467-018-03286-w)
Supplement: Supplementary file 1 — Supplementary Information [file 41467_2018_3286_MOESM1_ESM.pdf]

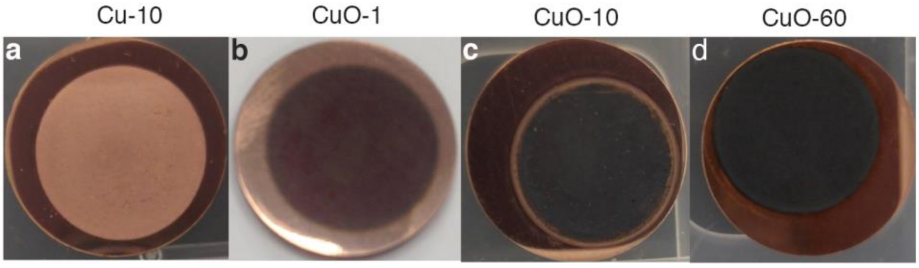

Supplementary Figure 1. Photographs of four as-deposited catalysts. The appearances of four catalysts before reduction: (**a**) Cu-10; (**b**) CuO-1; (**c**) CuO-10 and (**d**) CuO-60.

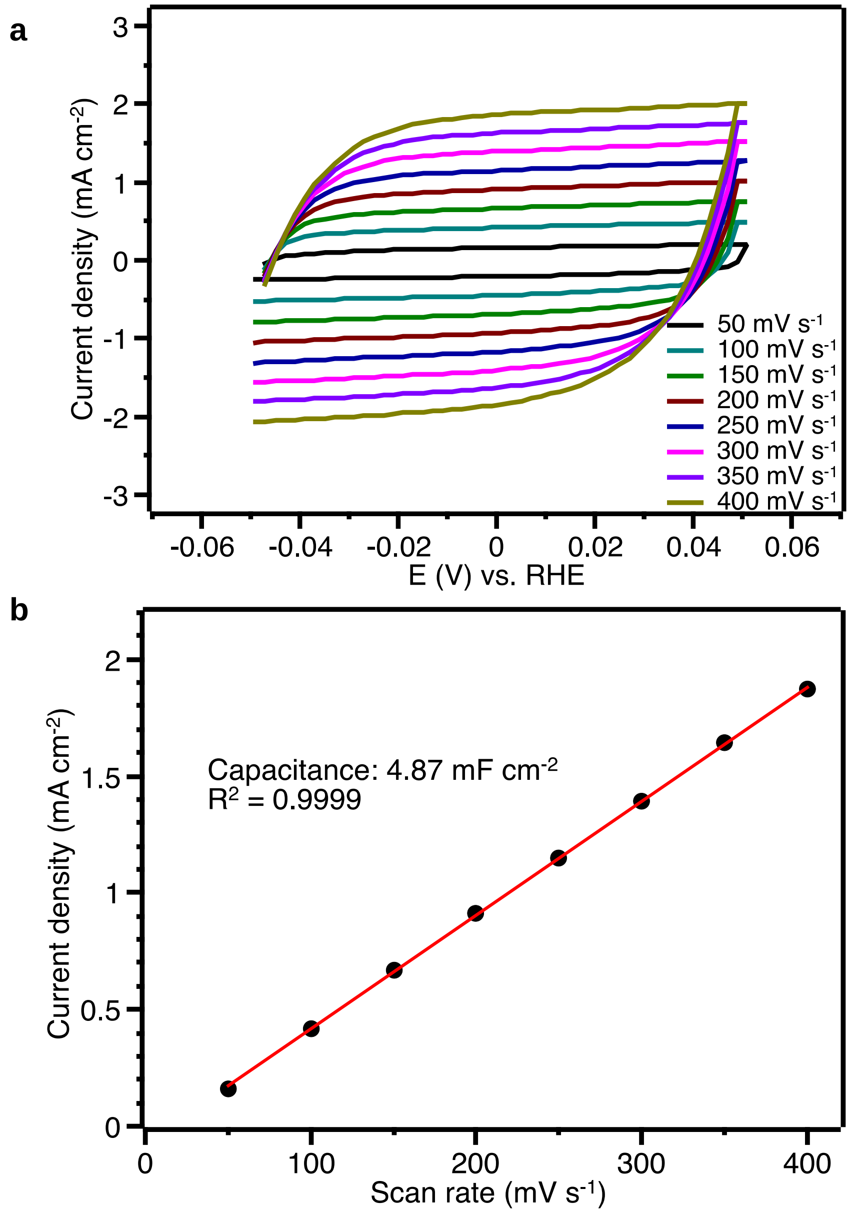

Supplementary Figure 2. Double layer capacitance measurements of CuO-60 after reduction. **a** Representative cyclic voltammograms at different scan rates from -0.05 V to 0.05 V vs. RHE on CuO-60 in 0.1 M $KClO_4$ (saturated with N_2) and **b** linear fitted curve of non-faradaic current density vs. scan rate.

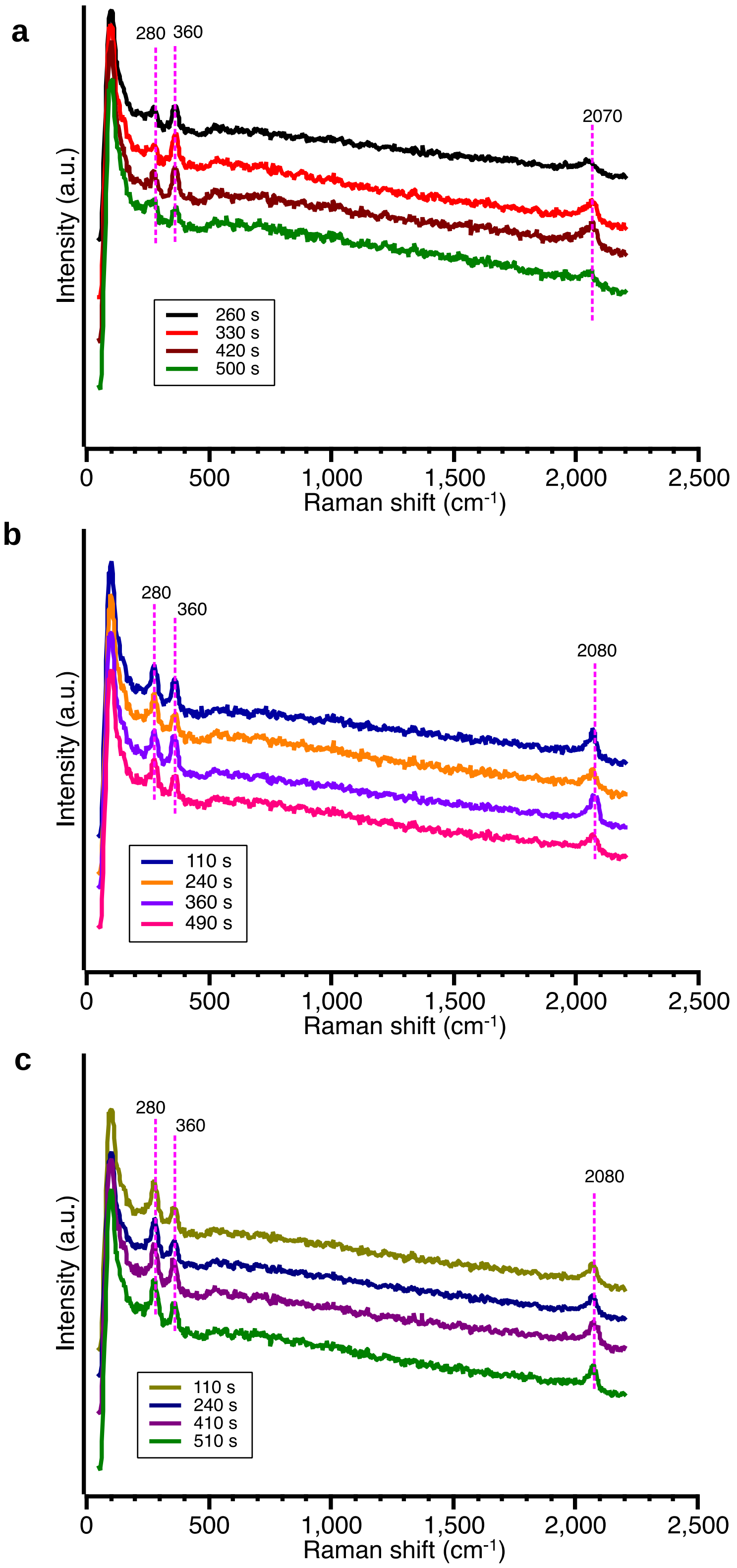

Supplementary Figure 3. Additional operando Raman spectra on CuO-60. Operando Raman spectra on CuO-60 at (**a**) -0.8 V, (**b**) -0.7 V and (**c**) -0.6 V vs. RHE.

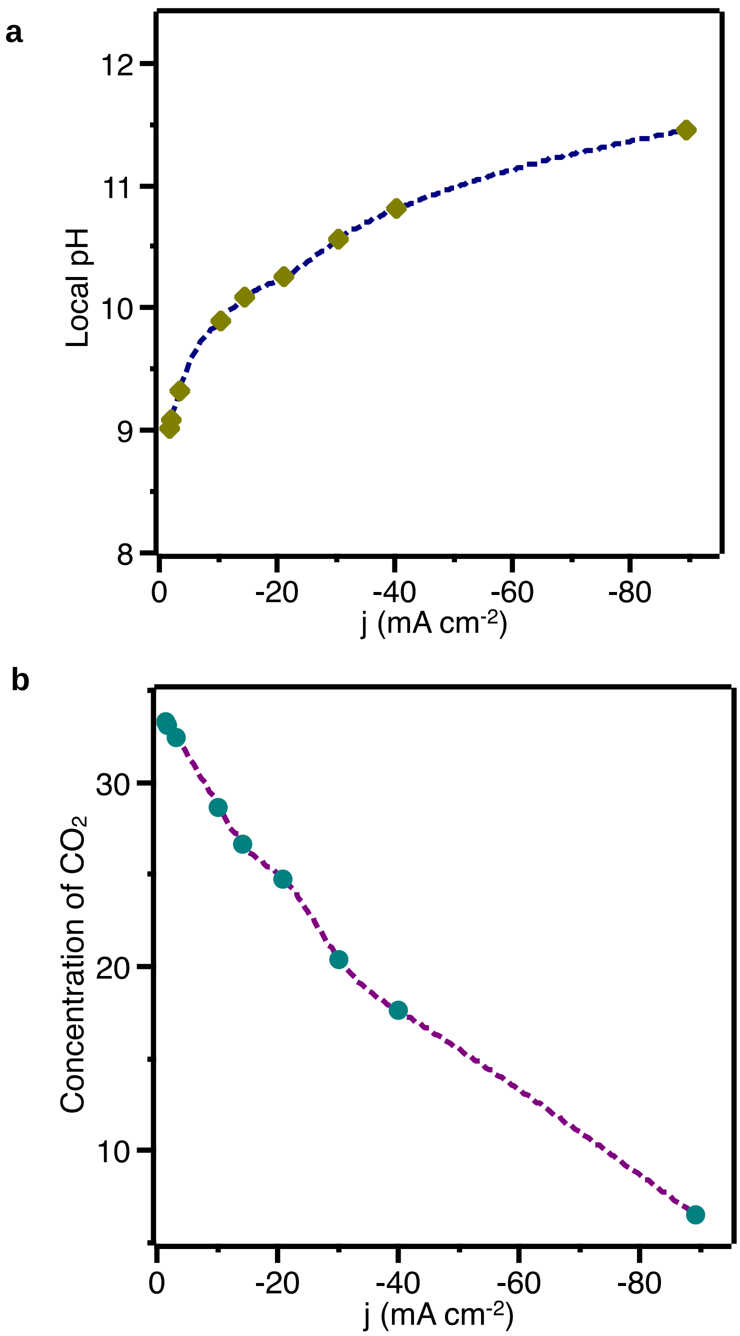

Supplementary Figure 4. Simulations of local pH and concentration of CO_2 using Cu-10 catalyst. **a** Simulated local pH values and **b** local concentration of CO_2 as a function of current density on Cu-10 catalyst.

Supplementary Table 1. Capacitance values (average of 3-4 separate measurements) and roughness factors of four catalysts after reduction. Roughness factor was calculated against the ideally smooth Cu surface (0.029 mF cm^{-2})[1].

Catalyst	Capacitance (mF cm^{-2})	Roughness factor
Cu-10	0.04	1.4
CuO-1	0.15	5
CuO-10	1.4	48
CuO-60	5.4	186

Supplementary Table 2. Faradaic efficiency of all the products quantified during CO_2 reduction using Cu-10 as the catalyst. CO – carbon monoxide, $HCOO^-$ – formate, CH_4 – methane, C_2H_4 – ethylene, C_2H_5OH – ethanol, H_2 – hydrogen, C_2H_6 – ethane, AD – acetaldehyde, PD – propionaldehyde, AT – acetone, AC – acetate, MO – methanol, PO – n-propanol, AA – allyl alcohol. "-"– not detected.

E (V)	j_o (mA cm^{-2})	CO	$HCOO^-$	CH_4	C_2H_4	C_2H_5OH	H_2	C_2H_6	AD	PD	AT	AC	MO	PO	AA	Total
-0.90	-1.75	14.95	25.25	0.43	1.10	0.16	61.67	-	0.24	0.46	-	-	-	0.39	0.17	104.82
-0.95	-2.12	10.40	23.85	1.80	5.31	1.42	54.40	-	1.10	2.06	-	0.56	-	0.88	0.97	102.75
-1.00	-3.42	9.36	10.72	9.36	15.90	3.41	42.35	-	2.57	3.07	0.03	0.95	-	1.96	1.78	101.46
-1.05	-10.37	2.52	6.41	25.53	27.52	8.79	23.31	-	3.14	2.13	0.13	1.21	0.05	2.62	2.01	105.37
-1.10	-14.55	3.16	3.76	38.59	19.34	7.98	24.23	-	3.04	1.28	0.10	1.59	0.14	1.63	1.37	106.21
-1.15	-21.14	0.75	3.37	54.31	7.48	3.54	24.31	-	0.89	0.23	0.06	0.67	0.10	0.42	0.34	96.47
-1.20	-30.43	0.46	1.38	62.25	4.20	2.22	31.37	-	0.49	0.09	0.04	0.31	0.06	0.18	0.14	103.19
-1.25	-40.27	0.14	1.07	43.87	1.16	0.55	55.54	-	0.13	0.05	0.05	0.11	0.03	0.08	0.05	102.83
-1.30	-89.38	0.01	0.37	5.29	0.14	0.23	90.21	-	-	-	0.01	-	-	0.07	-	96.33

Supplementary Table 3. Faradaic efficiency of all the products quantified during CO_2 reduction using CuO-1 as the catalyst. CO – carbon monoxide, $HCOO^-$ – formate, CH_4 – methane, C_2H_4 – ethylene, C_2H_5OH – ethanol, H_2 – hydrogen, C_2H_6 – ethane, AD – acetaldehyde, PD – propionaldehyde, AT – acetone, AC – acetate, MO – methanol, PO – n-propanol, AA – allyl alcohol. “-”– not detected.

E (V)	j_o (mA cm^{-2})	CO	$HCOO^-$	CH_4	C_2H_4	C_2H_5OH	H_2	C_2H_6	AD	PD	AT	AC	MO	PO	AA	Total
-0.90	-3.04	8.38	21.14	0.84	14.63	3.54	37.71	-	1.54	3.69	-	-	-	2.61	1.27	95.35
-0.95	-5.13	5.17	16.97	3.12	23.80	4.98	31.26	-	1.94	3.87	0.11	-	-	3.43	1.76	96.41
-1.00	-7.94	2.58	12.05	9.56	31.59	7.39	20.59	-	2.58	3.38	0.13	-	0.17	3.72	2.10	95.84
-1.05	-16.73	0.75	4.13	21.98	36.23	10.96	15.13	-	2.38	1.57	0.13	0.49	0.12	2.92	1.64	98.43
-1.10	-24.27	0.36	3.12	33.97	27.14	9.70	18.54	-	1.93	0.79	0.10	0.34	0.11	1.64	1.01	98.75
-1.15	-32.88	0.14	2.77	39.78	9.93	4.25	42.23	-	0.61	0.16	0.03	0.07	0.07	0.49	0.30	100.83
-1.20	-45.16	0.05	1.94	23.83	2.00	1.20	71.27	-	0.16	0.04	0.02	-	0.03	0.11	0.07	100.72

Supplementary Table 4. Faradaic efficiency of all the products quantified during CO_2 reduction using CuO-10 as the catalyst. CO – carbon monoxide, $HCOO^-$ – formate, CH_4 – methane, C_2H_4 – ethylene, C_2H_5OH – ethanol, H_2 – hydrogen, C_2H_6 – ethane, AD – acetaldehyde, PD – propionaldehyde, AT – acetone, AC – acetate, MO – methanol, PO – n-propanol, AA – allyl alcohol. "-"– not detected.

E (V)	j_o (mA cm^{-2})	CO	$HCOO^-$	CH_4	C_2H_4	C_2H_5OH	H_2	C_2H_6	AD	PD	AT	AC	MO	PO	AA	Total
-0.80	-9.86	5.68	15.91	0.01	14.74	4.56	53.68	1.11	0.58	1.85	0.11	0.65	0.13	4.72	0.50	104.23
-0.85	-14.90	2.62	11.64	0.04	24.10	7.26	44.45	0.71	0.63	2.25	0.17	0.68	0.15	6.20	0.87	101.77
-0.90	-21.96	1.49	9.24	0.12	31.02	10.69	35.49	0.61	0.59	1.48	0.18	0.56	0.11	5.72	0.99	98.29
-0.95	-31.50	0.46	4.02	0.28	33.79	14.22	38.21	0.17	0.65	0.81	0.17	0.33	0.07	4.23	0.93	98.34
-1.00	-40.06	0.20	1.79	0.51	29.32	12.57	52.78	0.07	0.57	0.39	0.09	0.16	0.06	2.20	0.55	101.26
-1.05	-52.44	0.06	0.72	1.15	15.89	6.86	76.17	-	0.30	0.14	0.04	0.08	0.05	0.88	0.25	102.59
-1.10	-97.91	0.09	0.13	0.87	2.79	0.56	98.24	-	0.03	0.01	-	0.02	0.01	0.05	0.02	102.82

Supplementary Table 5. Faradaic efficiency of all the products quantified during CO_2 reduction using CuO-60 as the catalyst. CO – carbon monoxide, $HCOO^-$ – formate, CH_4 – methane, C_2H_4 – ethylene, C_2H_5OH – ethanol, H_2 – hydrogen, C_2H_6 – ethane, AD – acetaldehyde, PD – propionaldehyde, AT – acetone, AC – acetate, MO – methanol, PO – n-propanol, AA – allyl alcohol. "-"– not detected.

E (V)	j_o (mA cm^{-2})	CO	$HCOO^-$	CH_4	C_2H_4	C_2H_5OH	H_2	C_2H_6	AD	PD	AT	AC	MO	PO	AA	Total
-0.45	-0.73	36.09	13.78	-	0.12	2.06	40.99	-	-	-	-	0.44	-	0.53	-	94.01
-0.50	-1.14	46.40	22.90	0.10	0.31	2.07	23.18	-	0.68	0.32	-	1.07	-	0.80	-	97.83
-0.55	-1.58	45.20	29.70	-	1.23	2.21	14.63	0.15	0.58	0.69	-	2.24	-	0.74	-	97.37
-0.60	-2.54	30.60	34.98	0.02	2.75	2.92	24.72	0.94	0.85	1.29	-	2.67	-	1.59	-	103.33
-0.65	-5.93	16.38	25.38	-	4.99	3.68	39.76	1.91	0.68	1.45	0.04	1.63	0.38	2.97	0.06	99.31
-0.70	-11.83	5.56	14.93	0.02	6.79	3.53	55.98	2.44	0.45	1.17	0.08	1.07	0.21	3.60	0.13	95.96
-0.75	-16.38	2.58	10.59	0.01	11.25	4.23	56.16	2.09	0.39	1.30	0.12	0.81	0.19	4.74	0.24	94.70
-0.80	-22.55	1.43	8.92	0.01	18.75	7.37	46.14	2.05	0.35	1.24	0.17	0.75	0.13	5.68	0.40	93.39
-0.85	-24.34	1.29	6.31	0.02	26.49	10.68	41.53	1.82	0.42	1.19	0.16	0.39	0.11	5.27	0.56	96.24
-0.90	-36.99	0.31	2.33	0.02	26.68	12.87	48.41	0.81	0.40	0.50	0.11	0.14	0.04	2.64	0.34	95.60
-0.95	-47.70	0.12	1.00	0.05	17.85	7.84	71.51	0.27	0.23	0.19	0.05	0.08	0.02	1.28	0.22	100.71
-1.00	-60.26	0.12	0.52	0.72	12.46	4.59	79.80	0.06	0.24	0.16	0.03	0.01	0.04	0.65	0.18	99.58

Supplementary Table 6. Selectivity of different Cu single crystals reported in literature. Electrolyte: 0.1 M $KHCO_3$.

Catalyst	Potential (V vs. RHE)	Selectivity	Author
Cu (111)	-1.15	44% CH_4, 3% C_2H_4	Huang et al.[2]
Cu (100)	-1.10	30% CH_4, 7% C_2H_4	Huang et al.[2]
Cu (100)	-0.95	26% C_2H_4, 6% CH_4	Huang et al.[2]
Cu (111)	-0.90	10% CO, 19% $HCOO^-$	Huang et al.[2]
Cu (110)	-0.80	25% $HCOO^-$	Huang et al.[2]
Cu (100)	-1.10	44% CH_4, 16% C_2H_4	Hahn et al.[3]
Cu (111)	-1.10	42% CH_4, 16% C_2H_4	Hahn et al.[3]
Cu (100)	-0.97	39% C_2H_4, 8% CH_4	Hahn et al.[3]
Cu (111)	-0.98	24% C_2H_4, 9% CH_4	Hahn et al.[3]
Cu (111)	-0.89	15% CO, 29% $HCOO^-$	Hahn et al.[5]

Supplementary Table 7. Selectivity of Cu catalysts reported in literature.

Catalyst	Electrolyte	Potential (V vs. RHE)	Current (mA cm^{-2})	Selectivity	Author
Isolated Cu nanoparticles	0.1 M $NaHCO_3$	-1.25	9	80% CH_4	Manthiram et al.[4]
Cu polycrystalline	0.1 M $KHCO_3$	-1.19	18	59% CH_4	Ren et al.[5]
Cu polycrystalline	0.1 M $KHCO_3$	-1.17	17	40% CH_4	Kuhl et al.[6]
3 C cm^{-2} Cu_2O	0.1 M $KHCO_3$	-1.10	37	33% C_2H_4	Kas et al.[7]
44 nm Cu cubes	0.1 M $KHCO_3$	-1.10	3	41% C_2H_4	Loiudice et al[8]
Cu nanoparticles	0.1 M $KHCO_3$	-1.05	16	30% C_2H_4	Ren et al.[9]
KF roughened Cu	0.1 M $KHCO_3$	-1.00	6	16% C_2H_4	Kwon et al.[10]
Electrochemically cycled Cu	0.1 M $CsHCO_3$	-1.00	14	45% C_2H_4	Lum et al.[11]
1.7 µm Cu_2O	0.1 M $KHCO_3$	-0.99	30	38% C_2H_4	Ren et al.[5]
Cu mesocrystal	0.1 M $KHCO_3$	-0.99	25	28% C_2H_4	Chen et al.[12]
Cu-NC10	0.1 M $KHCO_3$	-0.95	14	36% C_2H_4	Ren et al.[9]
Plasma activated Cu (O_2 20 W 2 min)	0.1 M $KHCO_3$	-0.92	18	60% C_2H_4	Mistry et al.[13]
CuO nanoparticles	0.5 M $KHCO_3$	-0.75	~ 3.5	61% $HCOO^-$	Gupta et al.[14]
Plasma activated Cu (O_2 20 W 2 min)	0.1 M $KHCO_3$	-0.65	1.7	55% CO	Mistry et al.[13]
Cu NC-20	0.1 M $KHCO_3$	-0.65	2	35% $HCOO^-$	Ren et al.[9]
CuO	0.1 M $KHCO_3$	-0.60	0.6	50% CO	Ma et al.[15]
Thick Cu_2O	0.5 M $NaHCO_3$	-0.55	2.6	39% $HCOO^-$	Li et al.[1]
11 C cm^{-2} Cu_2O	0.1 M $KHCO_3$	-0.40	~ 1	30% CO	Kas et al.[7]
Thick Cu_2O	0.5 M $NaHCO_3$	-0.35	0.6	47% CO	Li et al.[1]

Supplementary References

1 Li, C. W. & Kanan, M. W. CO_2 Reduction at Low Overpotential on Cu Electrodes Resulting from the Reduction of Thick Cu_2O Films. *J. Am. Chem. Soc.* **134**, 7231-7234, (2012).

2 Huang, Y., Handoko, A. D., Hirunsit, P. & Yeo, B. S. Electrochemical Reduction of CO_2 Using Copper Single-Crystal Surfaces: Effects of CO* Coverage on the Selective Formation of Ethylene. *ACS Catal.* **7**, 1749-1756, (2017).

3 Hahn, C. *et al.* Engineering Cu surfaces for the electrocatalytic conversion of CO_2: Controlling selectivity toward oxygenates and hydrocarbons. *Proc. Natl. Acad. Sci.* **114**, 5918-5923, (2017).

4 Manthiram, K., Beberwyck, B. J. & Alivisatos, A. P. Enhanced Electrochemical Methanation of Carbon Dioxide with a Dispersible Nanoscale Copper Catalyst. *J. Am. Chem. Soc.* **136**, 13319-13325, (2014).

5 Ren, D. *et al.* Selective Electrochemical Reduction of Carbon Dioxide to Ethylene and Ethanol on Copper(I) Oxide Catalysts. *ACS Catal.* **5**, 2814-2821, (2015).

6 Kuhl, K. P. *et al.* Electrocatalytic Conversion of Carbon Dioxide to Methane and Methanol on Transition Metal Surfaces. *J. Am. Chem. Soc.* **136**, 14107-14113, (2014).

7 Kas, R. *et al.* Electrochemical CO_2 Reduction on Cu_2O-derived Copper Nanoparticles: Controlling the Catalytic Selectivity of Hydrocarbons. *Phys. Chem. Chem. Phys.* **16**, 12194-12201, (2014).

8 Loiudice, A. *et al.* Tailoring Copper Nanocrystals towards C_2 Products in Electrochemical CO_2 Reduction. *Angew. Chem. Int. Ed.* **55**, 5789-5792, (2016).

9 Ren, D., Wong, N. T., Handoko, A. D., Huang, Y. & Yeo, B. S. Mechanistic Insights into the Enhanced Activity and Stability of Agglomerated Cu Nanocrystals for the Electrochemical Reduction of Carbon Dioxide to *n*-Propanol. *J. Phy. Chem. Lett.* **6**, 20-24, (2016).

10 Kwon, Y., Lum, Y., Clark, E. L., Ager, J. W. & Bell, A. T. CO_2 Electroreduction with Enhanced Ethylene and Ethanol Selectivity by Nanostructuring Polycrystalline Copper. *ChemElectroChem* **3**, 1012-1019, (2016).

11 Lum, Y., Yue, B., Lobaccaro, P., Bell, A. T. & Ager, J. W. Optimizing C–C Coupling on Oxide-Derived Copper Catalysts for Electrochemical CO_2 Reduction. *J. Phys. Chem. C* **121**, 14191-14203, (2017).

12 Chen, C. S. *et al.* Stable and Selective Electrochemical Reduction of Carbon Dioxide to Ethylene on Copper Mesocrystals. *Catal. Sci. Technol.* **5**, 161-168, (2015).

13 Mistry, H. *et al.* Highly Selective Plasma-Activated Copper Catalysts for Carbon dioxide Reduction to Ethylene. *Nat. Commun.* **7**, 12123, (2016).

14 Gupta, K., Bersani, M. & Darr, J. A. Highly efficient electro-reduction of CO_2 to formic acid by nano-copper. *J. Mat. Chem. A* **4**, 13786-13794, (2016).

15 Ma, M., Djanashvili, K. & Smith, W. A. Selective electrochemical reduction of CO_2 to CO on CuO-derived Cu nanowires. *Phys. Chem. Chem. Phys.* **17**, 20861-20867, (2015).
